# Microsurgical dissection of Sylvian fissure—short technical videos of third generation cerebrovascular neurosurgeons

**DOI:** 10.1007/s00701-019-03999-x

**Published:** 2019-07-08

**Authors:** Sajjad Muhammad, Rokuya Tanikawa, Michael Lawton, Luca Regli, Mika Niemelä, Miikka Korja

**Affiliations:** 1 Department of Neurosurgery, University of Helsinki and Helsinki University Hospital, Helsinki, Finland; 2 Department of Anatomy and Developmental Biology, Centre for Biomedicine and Medical Technology Mannheim (CBTM) and European Center for Angioscience (ECAS), Medical Faculty Mannheim, Heidelberg University, Mannheim, Germany; 3 Teishinkai Hospital, Sapporo, Japan; 4 Barrow Neurological Institute, Phoenix, AZ USA; 5 University Hospital Zürich, Zürich, Switzerland

**Keywords:** Sylvian fissure, Sharp dissection, Blunt dissection, Dissection styles

## Abstract

**Background:**

Multiple intracranial pathologies, including aneurysms of the middle cerebral artery, distal basilar artery, and suprasellar pathologies require the microsurgical opening of the Sylvian fissure. Delicate splitting of the arachnoid and safe microdissection of the veins, arteries, and brain parenchyma is the key to successful surgery through the Sylvian fissure corridor. We hypothesize that the geographical and historical environment in which neurosurgeons learn their operative skills is subject to a number of extrinsic influences, including cultural nuances of surgical techniques. Here we try to illustrate some cultural differences and technical aspects of the opening of the Sylvian fissure by four “third generation” cerebrovascular neurosurgeons from three different continents.

**Methods:**

In the video analysis, various microsurgical aspects, including the opening style of the Sylvian fissure, handedness, use of sharp or blunt microinstruments, use of retractors, use of high magnification, and handling of bridging veins are presented.

**Results:**

The video illustrates the two distinct Sylvian fissure opening styles, namely sharp and blunt microdissection, as well as the extent of the opening namely a wide and focal splitting.

**Conclusion:**

The edited video underlines nuances and differences of a few major technical aspects that are perhaps typical to certain surgical environments and cultures. These microsurgical nuances and styles are useful pearls that can be mastered with training by any novice neurosurgeon.

**Electronic supplementary material:**

The online version of this article (10.1007/s00701-019-03999-x) contains supplementary material, which is available to authorized users.

## Introduction

The Sylvian fissure (SF), i.e., the lateral sulcus, separates the frontal and parietal lobes from the temporal lobe. The insular cortex and main branches of the middle cerebral artery (MCA) are covered by the opercula and located deep in the SF. Since the introduction of the microscope in neurosurgery [2], the opening of the SF has become an indirect measure of microneurosurgical competence. Professor Yasargil established in Zurich the school of modern microneurosurgery and became the master of the SF. Already in 1969, Yasargil and Fox embraced a keyhole craniotomy concept and the use of high magnification [5]. Soon after, Yasargil and colleagues launched the concept of using “natural spaces,” such as the SF, in microsurgical approaches [4]. The “natural spaces” concept meant that following the CSF spaces and the arachnoid cisterns avoiding manipulation of the brain parenchyma to reach the surgical lesion emerged as one of the most important microneurosurgical techniques.

After Professor Yasargil and colleagues popularized the concept of cisternal microsurgery, numerous technical variations and nuances have been introduced by a vast number of neurosurgeons all over the world. In Europe, Professor Hernesniemi from the Helsinki University Hospital refined the Yasargil approach to its greatest possible potential and efficacy [1]. In Asia, Japanese Professors Sugita, Fukushima, and Kamiyama contributed strongly to the modern “Eastern way” of cisternal surgery. In North America, perhaps the strongest influence on the SF opening styles emerged from the school of Professor Spetzler. As many successors follow the example of renowned surgeons, and since surgical training at prominent departments often consists of cultural demands and characteristics, certain surgical “signatures” may perhaps be proliferated identified.

We qualitatively evaluated the opening of the Sylvian fissure by “3rd generation” neurosurgeons from three continents and four different nationalities. We hypothesized that we could identify some cultural and historical “signatures” that are perhaps typical to a certain school of thought. Moreover, as we illustrate some differences in techniques of microdissection of the arachnoid and splitting of the Sylvian fissure, the description of these microsurgical nuances may represent a precious help to master the opening of the Sylvian fissure for young neurosurgeons in training.

## Methods

We present four short and edited videos of various microneurosurgical styles and technical nuances of the Sylvian fissure dissection. In all videos, the approach was used to clip an unruptured middle cerebral artery aneurysm. The aim of the short video clips is to visualize some differences in microsurgical dissection technique among four cerebrovascular neurosurgeons. All surgeries were uneventful and performed in 2018. All but video 1A were recorded during the Helsinki Live Course 2018. All surgeries were uneventful and performed in 2018.


Video 1Demonstration of different styles of dissection of the SF to clip an unruptured MCA aneurysm. (MP4 344664 kb)


We focused on the following aspects in the SF opening: overall opening style of the SF, handedness, and use of sharp microinstruments, use of blunt microinstruments, use of retractors, use of magnification, handling of bridging veins, use of cottonoids/patties, and use of other known techniques, such as the water dissection technique of Toth [3]. The opening style of the SF can perhaps be divided into two main approaches, namely blunt and sharp dissection. In blunt dissection, not only sharp instruments but also blunt instruments (most often bipolars) are used in the opening of the fissure. Moreover, the blunt or sharp opening can be further divided into a wide and focused opening.

## Results

### General

One out of four surgeons used the sharp dissection style, meaning that he cut the arachnoid with microscissors*.* The other three performed the dissection using repeatedly or at least occasionally the tips of a bipolar. Three out of four surgeons opened the SF widely. Only one surgeon used the highly focused SF opening (approximately 1 cm, focused at the area of the lesion). Three out of four surgeons were right-handed. All used a high magnification during the dissection. Suction was held in the non-dominant hand by all. Only one out of four surgeons did not use surgical cottonoids to protect and cover the brain tissue. Special care was taken to save the Sylvian and bridging veins by all surgeons. One surgeon dissected and preserved even small veins under high magnification. The water dissection technique of Toth was used by one surgeon (not shown in the video).

### Master videos

#### Video 1A

A right-handed surgery is performed using the sharp dissection approach with microscissors. Retractors are used to ease the sharp dissection of the arachnoid membranes and adhesions with Kamiyama microscissors under proper visualization at high magnification (× 15). Kamiyama microscissors have exceptionally sharp blades that allow membrane cutting at the very tip with consistent cutting efficiency. In this sharp dissection style, blunt dissection with bipolars is used very seldomly or not at all. Special care is taken to preserve each and every vein. Small arteries are often freed from any adherence and moved away, thus allowing better access to the lesion. The SF is opened widely, and the banks of the fissure are covered with cottonoids. The microscope is controlled with foot pedals.

#### Video 1B

A left-handed surgery is performed using predominantly the sharp dissection style, but bipolar forceps are occasionally used in opening the fissure. Therefore, this style is classified as the blunt opening style. Retractors are not used, as the suction serves as a retractor along the opening. Microscissors (particularly the tips) are used to cut the arachnoid layer under high magnification (× 12). No cottonoids are used and the SF is opened widely. In this video, the microscope is manually controlled but usually done with a mouthpiece.

#### Video 1C

A right-handed surgery is performed using both the sharp and blunt dissection style (the latter used even more). Retractors are not used at all and the suction tube is used as a dynamic retractor. Toth’s water dissection is often used to clean the trabecular tissue and facilitate the dissection (not shown in the video). The SF is opened under high magnification (× 12) using the tips of the blunt bipolar forceps. Cottonoids are used to cover the brain during the dissection. A highly focused opening (approximately 1 cm) of the SF is performed to reach the lesion. The microscope is controlled by the mouthpiece. The surgeon was operating on standing up with arm rest.

#### Video 1D

A right-handed surgery is performed using mainly sharp but also blunt dissection. The superficial arachnoid layer is opened parallel to the fissure with a 23 G needle tip. The SF is further opened using predominantly sharp dissection at high magnification (× 10). Retractors are not used at all as the suction serves again as a dynamic retractor. In the depth, blunt dissection with bipolar tips is occasionally performed. The SF is opened widely to achieve maximum exposure. Microscope is moved using the mouthpiece.

## Discussion

In different countries and continents, neurosurgeons are likely to follow the style and technique of the centers where they were trained. We identified some surgical “signatures” that are perhaps typical in a certain culture and environment. In the years following the 2nd World War, one Japanese philosophy, namely Kaizen, started to gain international popularity in multiple industries from business to healthcare. In Kaizen, a dedication to seek continuous improvements will add up to a substantial change in effectiveness, satisfaction, and outcome over time. An example of the Japanese dedication and mastery is the training to become a respected sushi chef—the training will take precision and commitment of more than 10 years. In Japanese neurosurgery, the mastery of technical skills is typically striking. In contrast, post-industrial working-time regimes have shaped the work-life balance especially in the Northern Europe. Most characteristically, legal restrictions of working hours have led for example in neurosurgery to an increased work intensity and performance figures. In such circumstances, minimal and simple adjustments that are likely to increase productivity gain often popularity.

The videos showed particularly the two distinct styles of the SF opening (video 1a–d), namely the Western and Eastern styles. Professor Yasargil is perhaps the most well-known surgeon who used the blunt Western dissection style. Especially European neurosurgeons, who often come from the “school of Yasargil,” use bipolar tips most of the time to bluntly dissect the arachnoid membranes. The Western technique of bipolar tearing of the arachnoid and creating space between the temporal and frontal lobes has been used in three generations (GM Yasargil, J Hernesniemi, M. Niemelä/L. Regli/M. Lawton). Splitting the fissure with the tips of bipolar must be learned properly, as the style easily causes arachnoidal and even cortical microbleeds, which are perhaps observed less often in the sharp Eastern dissection style. In particular, Professor Hernesniemi used the highly focused opening to avoid any unnecessary trauma to neurovascular structures, the risk of which increases with wider openings. In aneurysmal SAH, the blunt Western dissection style may often be safer and is much faster, as blood clots in the SF make sharp dissection overly challenging. As the fissure is split very shortly (focused), some minor cortical traumas may occasionally occur. This style is well adopted by Professor Niemelä (video 1c) from the Helsinki University Hospital. The focused opening of the Western style, however, requires perhaps more experience and a thorough 3-dimensional understanding of the anatomy, especially if no navigation is used.

In the sharp Eastern dissection style, which is particularly common among Asian (video 1a) neurosurgeons, but used also by American neurosurgeons (video 1b), microscissors or a knife is consistently used. In this style, not only the suction but also the sharp instrument itself can be used as a retractor and to put tension on the arachnoid membranes. However, surgeons that use the sharp dissection style tend infrequently to use retractors (video 1a) instead of a technically more demanding “bimanual retraction” approach. In principle, retractors should not be used today to retract the brain but only to hold the brain in one position. Whether the Western blunt or Eastern sharp dissection leads to better preservation of venous, arterial, and neural structures is unclear, especially since postoperative magnetic resonance imaging studies and thorough neuropsychological examinations are not routine in most departments. Our understanding is, however, that the focused blunt opening of the SF is somewhat faster than the perhaps more elegant sharp dissection (video 1a–d). In any case, in the presence of bridging veins, Eastern sharp dissection style seem to handle and detach the veins more easily and safely than so-called “blunt dissectors.” Irrespective of dissection style, all “third generation” cerebrovascular neurosurgeons used high magnification (at least × 10) to properly visualize the neurovascular structures.

Our qualitative video analysis has numerous shortcomings. Even though none of the operators was involved in the video analysis, it is likely that the authors avoided presenting the descriptive results and video clips in an overly critical light. Moreover, dividing the surgical techniques into two distinct categories on the basis of a Sylvian fissure splitting is not perhaps justified. Nevertheless, the article tries to underline the fact that geopolitical and cultural differences exist in neurosurgery, and that technical differences between well-known cerebrovascular neurosurgeons are surprisingly striking (even in the early phases of complex cerebrovascular surgeries). For younger neurosurgeons, it may be of importance to realize that no one truth exists. In fact, future neurosurgeons should evolve from the previous generations and combine the best characteristics of their teachers and role models. Last, our sample of surgeons is not representative, and only by analyzing numerous neurosurgeons from various continents could perhaps give a more reliable picture of cultural differences. However, the authors have seen techniques of hundreds of surgeons from all continents, apart from Antarctica, and believe that the made interpretations are somewhat representative.

Overall, dissection of the arachnoid membrane can be achieved in various styles, and every style needs proper and continuous training irrespective of the training center. Generally, a focused opening (around 1 cm) can be safely performed with bipolar tips but for a wide opening of the whole Sylvian fissure, perhaps the sharp dissection with microscissors is more elegant and safer. For young neurosurgeons, it is important to be flexible and to train in various technical aspects to ensure safe dissection of the SF. Therefore, we encourage neurosurgeons to travel more and to learn from fellow neurosurgeons.

## Conclusion

Both styles of splitting the SF can be performed safely. While a sharp dissection under high magnification with more cutting and less splitting may reduce minor bleeds, this is perhaps a somewhat more laborious approach. Technical aspects of both styles require training but can be adapted by any novice neurosurgeon.
